# Supplementary material for: Evaluation of a successful fluoroquinolone restriction intervention among high-risk patients: A mixed-methods study
Source: PLoS One. 2020 Aug 25;15(8):e0237987. doi: 10.1371/journal.pone.0237987 (PMC7446965; doi:10.1371/journal.pone.0237987)
Supplement: S1 Appendix — (DOCX) [file pone.0237987.s001.docx]

**Semi-structured interview guide: Fluoroquinolone Usage in Intensive Care and Post-Transplant Care Units**

Study purpose: The purpose of this study is to determine the perceptions of healthcare workers regarding patterns of fluoroquinolone usage and the potential adverse and desirable effects of fluoroquinolone usage

**Topic Area I: Background and Contextual Issues**

What is your title/position?

What degree(s) do you hold?

What specialty training have you done?

How many years of experience do you have of training?

How long have you been at this institution?

Can you describe the responsibilities of your position?

What tasks do you perform on a daily basis?

**Topic Area II: Perceptions of fluoroquinolone usage and CDI prevention**

What is your perception of the current culture on your unit regarding the use of fluoroquinolone? **(organization)**

What indications are routinely used to justify FQ usage?

Which FQ are used for these indications?

What are the expectations from staff regarding use?

In your opinion, how frequently are fluoroquinolones selected over alternative, similarly efficacious antimicrobials? How often do you think FQL are used inappropriately?

What factors encourage FQL use?

What factors discourage FQL use?

Which types of individuals on your team are most involved with antibiotic selection?

What sort of variability amongst providers have you encountered with regard to antibiotic selection? **(person)**

How frequently do you see providers using the antibiogram to guide antibiotic selection?

How often do you personally use the antibiograms? How often do you refer others to the antibiograms?

How frequently do you take into account an antibiotics ability to select for or against *C. diff*?

How frequently do you think other providers consider an antibiotics ability to select for or against *C. Diff*?

If the antibiogram included information about which antibiotics predispose to *C. diff*, how would that change your prescribing practices?

What is your perception of the hospital administration’s priorities regarding antibiotic stewardship, particularly with regards to *C. diff* prevention? **(organization)**

What role do provider-led groups (M.D., R.Ph.) play in effecting these priorities?

What opportunities do you know of for leadership (provider or non-medical) to discuss ideas regarding evidence-based selection of antibiotics with providers?

Can/does leadership support and promote these changes and how?

Can you recall initiatives in the past that attempted to reduce the prescribing of particular antibiotics, including FQL? Were any of these directed at *C. diff* prevention?

What roles do different team members play with decisions regarding the cessation/switching of an antibiotic (e.g., the role of pharmacists, attendings, etc.) **(person)**

Who could take on more/less responsibility?

**Topic Area III: Current Practices**

Regarding your practice for prescribing antibiotics for patients admitted to (unit):

For which indications do you routinely prescribe antibiotics?

How frequently is antibiotic selection discussed within your care team?

*Pharmacists:* How do you communicate with MD providers about antibiotic options?

*MDs:* In what situations would you seek out a pharmacist to help with antimicrobial decision making?

What tools or resources do you consult to determine which antibiotic(s) to select? **(tech and tools)**

What processes are in place to facilitate antibiotic selection (for particular indications)? –reminders, algorithms, order sets **(tech and tools)**

Regarding antibiotic selection decisions - **(task)**

Which agents do you use most frequently, and for which indications?

How frequently does a patient’s personal history of CDI affect your antibiotic selection? **(task)**

Is predisposition to *C. diff* a topic you discuss with patients if they had a history of *C. diff*? **(person)** How can/could these conversations be beneficial/harmful?

How do you make the decision to stop or switch antibiotics? **(task)**

What processes are in place to facilitate discontinuation or class switching? **(tech and tools)**

How does a pending discharge affect your decisions regarding antibiotic selection?

**(person)**

*If perception of overprescription or not enough attention to CDI:*

What can we do to change FQL prescribing practices?

What are advantages of FQ over other antibiotics?

What are disadvantages of FQ over other antibiotics?
