# Supplementary material for: Evaluation of a successful fluoroquinolone restriction intervention among high-risk patients: A mixed-methods study
Source: PLoS One. 2020 Aug 25;15(8):e0237987. doi: 10.1371/journal.pone.0237987 (PMC7446965; doi:10.1371/journal.pone.0237987)
Supplement: S1 Table — (DOCX) [file pone.0237987.s002.docx]

| **eTable 1. Indications for antimicrobials on study units, one month post-fluoroquinolone restriction policy implementation, by pharmacy review.** | | | |
| --- | --- | --- | --- |
|  | Cases, total | Cases, adherence to policy | % adherent to policy |
| Transplant Unit | | | |
| Positive urine culture in deceased kidney donor | 3 | 3 | 100 |
| Cystitis in renal transplant recipient | 2 | 2 | 100 |
| Pyelonephritis in renal transplant recipient | 2 | 2 | 100 |
| Cholangitis in liver transplant recipient | 2 | 2 | 100 |
| ICU | | | |
| Septic shock, unknown source | 2 | 3 | 66.6 |
| Septic shock, urinary source | 3 | 1 | 33.3 |
| Transplant Unit and ICU, combined | | | |
| Intraabdominal infection | 9 | 7 | 77.8 |
| Community acquired pneumonia | 6 | 6 | 100 |
| Hospital acquired pneumonia | 7 | 4 | 57.1 |
| Other* | 15 | N/A | |
| Total infections | 52 | 44 | 84.6 |
| *Adherence not able to be assessed, institutional policy did not indicate fluoroquinolone alternatives for all infectious syndromes | | | |
